# Antiplatelet Drugs on the Recurrence of Hepatocellular Carcinoma after Liver Transplantation

**DOI:** 10.3390/cancers14215329

**Published:** 2022-10-29

**Authors:** Mun Chae Choi, Eun-Ki Min, Jae Geun Lee, Dong Jin Joo, Myoung Soo Kim, Deok-Gie Kim

**Affiliations:** Department of Surgery, The Research Institute for Transplantation, Yonsei University College, Seoul 03722, Korea

**Keywords:** liver transplantation, antiplatelet, hepatocellular carcinoma, thromboprophylaxis

## Abstract

**Simple Summary:**

Recently, antiplatelet agents have been shown to have anticancer effects, especially for hepatocellular carcinoma (HCC) but have never been studied in liver transplantation (LT) recipients. We investigated 468 LT patients to ensure that antiplatelet drugs (aspirin or clopidogrel) could prevent HCC recurrence after LT. In matched patients, the 5-year incidence of HCC recurrence (15.8% versus 20.4%) and death from HCC (18.3% versus 15.4%) were not significantly different between the antiplatelet and non-antiplatelet groups. When adjusted for other risk factors of HCC recurrence, antiplatelet use was not associated with HCC recurrence. Unlike in non-recipients with liver disease, antiplatelet therapy did not affect HCC recurrence or death from HCC when used after LT.

**Abstract:**

Previous studies reported suppressive effects of antiplatelet agents on hepatocellular carcinoma (HCC); however, this has never been assessed in patients who underwent liver transplantation (LT). This retrospective observational study used data from LT recipients with pre-transplant HCC in a single tertiary hospital. The study population was divided into two groups according to the use of antiplatelet agents for >90 days within the study period (377 antiplatelet groups versus 91 non-antiplatelet groups). Matched groups containing 79 patients in each group were also compared regarding HCC-recurrence and HCC-related mortality, which were analyzed by treating non-HCC death as a competing risk. In Kaplan–Meier analyses of the matched cohort, the 5-year cumulative incidences of HCC recurrence and HCC-specific death were similar between the antiplatelet (*p* = 0.876) and non-antiplatelet groups (*p* = 0.701). All-cause and non-HCC deaths were also similar between the two groups (*p* = 0.867 and *p* = 0.413, respectively). In multivariable analyses of the entire cohort, antiplatelet use was not associated with HCC recurrence (hazard ratio [HR] 1.37, *p* = 0.300) or HCC-specific death (HR 1.54, *p* = 0.310). Therefore, unlike the usual setting with liver disease, antiplatelet therapy did not affect HCC recurrence or HCC-specific mortality when used after LT.

## 1. Introduction

Liver transplantation (LT) is an optimal treatment option for unresectable hepatocellular carcinoma (HCC) that can simultaneously cover underlying liver diseases. However, 10–15% of overall recurrence after LT [1] and the expansion of LT candidates, owing to the development of a downstaging strategy [2] have focused on reducing the recurrence of HCC after LT. 

Recent studies reported that higher platelet counts negatively affect the prognosis of patients with HCC [3,4]. Platelets have been known to play an important role in the survival and spread of tumor cells [5,6]. Based on these results, there have been several studies on the association between antiplatelet therapy and HCC. Sitia et al. [7] and Lee et al. [8] reported that antiplatelet drugs reduced HCC development in patients with hepatitis B virus (HBV). A more recent national population-based study revealed that low-dose aspirin was associated with a significantly lower rate of HCC in patients with chronic hepatitis without an increased risk of bleeding [9]. 

In patients diagnosed with HCC, antiplatelet therapy has been reported to be effective in increasing survival [10]. Furthermore, a population-based study reported that antiplatelet therapy could reduce HCC recurrence after resection in HBV-infected patients [11]. However, the possible antitumor effect of antiplatelet therapy has never been researched in LT patients, although a higher platelet count before LT has been reported to be associated with a lower recurrence of HCC after LT [12]. 

Antiplatelet drugs have been used after LT for thromboprophylaxis to reduce hepatic artery thrombosis [13,14] although there is no definite indication resulting in variability of antiplatelet use among LT recipients with HCC. Thus, we investigated the effect of antiplatelet drugs on the outcomes of patients with HCC who underwent LT. 

## 2. Materials and Methods

### 2.1. Study Population

**Figure 1:**
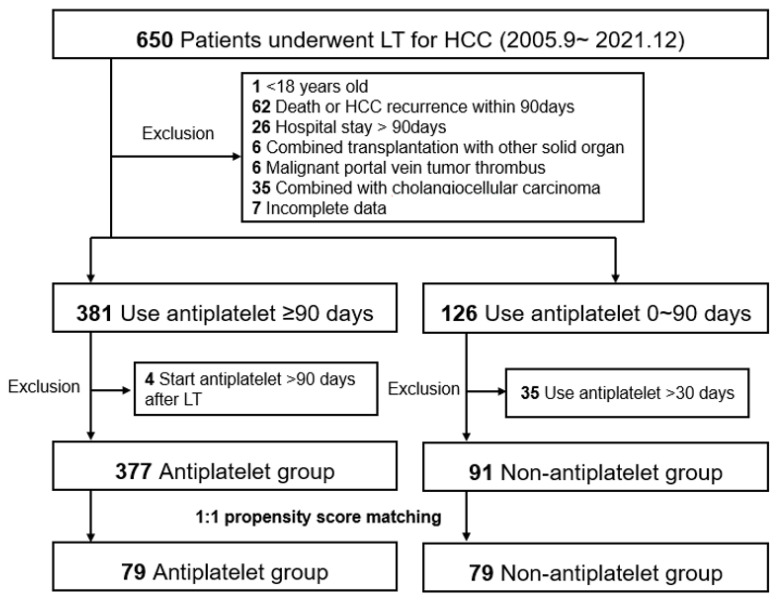
Study population. The flowchart diagram shows the patient selection, groupings, exclusion criteria, and propensity score matching cohorts. HCC, hepatocellular carcinoma; LT, liver transplantation. HCC, hepatocellular carcinoma; LT, liver transplantation.

We performed a retrospective single-center observational study using data from patients with HCC who underwent LT between September 2005 and December 2021 at Severance Hospital, Korea. The study end date of each patient was set at patient death, loss to follow-up, or 30 June 2022, whichever came first. Among the 650 study candidates, the following exclusion criteria were applied: (1) age < 18 years, (2) patient death or HCC recurrence within 90 days, (3) hospital stay > 90 days (excluding patients who were prone to death and not suitable for antiplatelet therapy owing to initial deterioration after LT), (4) combined transplantation with other solid organs, (5) malignant portal vein tumor thrombus, (6) combined with cholangiocellular carcinoma, and (7) incomplete data. We then divided the patients into two groups according to the use of antiplatelet drugs for >90 days before HCC recurrence within the study period. Patients who started antiplatelet therapy >90 days after LT were excluded from the antiplatelet group (*n* = 4). Patients who used antiplatelet drugs for >30 days were excluded from the non-antiplatelet group (*n* = 35). From the entire cohort of 377 antiplatelet and 91 non-antiplatelet groups, we established a 1:1 matched cohort with propensity scores (*n* = 79 in both matched groups, Figure 1). Both entire and matched cohorts were included in the analysis.

### 2.2. Data Collection

We recorded the baseline data of eligible LT patients from our electronic medical records, in addition to recording donor characteristics and transplantation factors. Factors associated with the risk of HCC recurrence were recorded, including salvage LT, pre-transplant bridging therapy, explant pathology, and tumor markers (AFP and PIVKA-II). Hospital stay was recorded as the duration from LT to the first discharge in days. Immunosuppressants were categorized according to the regimen used 3 months after LT: tacrolimus, tacrolimus+mycophenolate, and tacrolimus+mTOR inhibitor. As the ordinary target trough level of tacrolimus was 5~8 ng/mL during the first month, and 4~6 thereafter in our institution, we compared serial values of it throughout the study period between the antiplatelet users and non-antiplatelet users.

To compare whether antiplatelet therapy was indicated by platelet volume and to evaluate the inflammation score associated with tumor recurrence, the platelet count, platelet-lymphocyte ratio, and neutrophil-lymphocyte ratio immediately before LT were recruited. Laboratory results indicating liver function, such as AST, ALT, total bilirubin, and INR, were recorded 1 month after LT.

### 2.3. Outcomes

The primary outcomes were HCC recurrence and HCC-specific death. HCC recurrence was defined as the first notification of recurrent HCC in imaging studies that were screened at least every 3 months for the first year post-transplantation, at 3–6 month intervals until 2 years, and then annually. HCC-specific death was defined as death after HCC recurrence. The secondary outcomes were all-cause and non-HCC death. Non-HCC death was defined as death without HCC recurrence after LT.

### 2.4. Statistical Methods

Data are presented as median (interquartile range [IQR]) for continuous variables according to their normality and number (proportion) for categorical variables. For group comparison in the entire cohort, the Mann–Whitney U test and χ2 test were used when appropriate. For matched analyses, propensity score matching was performed using the nearest neighbor method with a caliper of 0.2 [15]. To generate the propensity score, all available baseline variables were used regarding the absolute standardized mean difference under 0.2 as the upper limit of balance after matching. Kaplan–Meier curve analyses were performed to compare outcomes, such as HCC recurrence, HCC-specific death, all-cause death, and non-HCC death, between the matched groups using the log-rank test. Multivariate Cox regression analysis was performed to assess the independent effect of antiplatelet therapy in the entire cohort. Covariates included in the multivariate models were selected using a stepwise method. Non-HCC death was treated as a competing risk when analyzing HCC recurrence and HCC-specific death, and HCC death was treated as a competing risk when analyzing non-HCC deaths. Competing risk regression was conducted according to the Fine and Gray method [16]. All analyses were performed using the R statistical package, version 4.2.0, for macOS (http://cran.r-project.org/) (Accessed on 1st Feburary, 2022), with the threshold for significance set at *p* < 0.05.

## 3. Results

### 3.1. Baseline Characteristics and Laboratory Results

In the entire cohort, most baseline characteristics were similar between the antiplatelet and non-antiplatelet groups, except for pre-transplant MELD and hospital stay (Table 1). Liver graft function and renal function 1 month after LT were similar between the two groups, although the INR was slightly higher in the non-antiplatelet group than in the antiplatelet group. After matching, we ensured that the absolute standardized mean difference for all variables was below 0.2 in all variables indicating adequate matching. Immunosuppressant regimens were similar between matched groups. Trough levels of tacrolimus were also similar throughout the study period (Figure S1).

In the entire antiplatelet group, patients started antiplatelet agents at a median of 7 (IQR, 7–8; minimum, 1; maximum, 67) days after LT. The subclasses of antiplatelet drugs were aspirin (*n* = 357), clopidogrel (*n* = 3), and dual antiplatelet (*n* = 17). Reasons for the use of dual antiplatelet were related with cardiovascular disease (*n* = 6), need for more potent thromboprophylaxis due to compromised anastomosis of hepatic vein/vena cava (*n* = 4), hepatic artery (*n* = 2), portal vein (*n* = 1), and unclear reason (*n* = 3). The median duration of antiplatelet drug use was 613 (IQR, 346–1776; minimum, 94; maximum, 1825) days. In the matched antiplatelet group, patients started antiplatelet agents at a median of 7 (IQR, 7–8; minimum, 1; maximum, 67) days after LT. The antiplatelet drugs were aspirin (*n* = 74) and dual antiplatelet agents (*n* = 5). The duration of antiplatelet drug use was 520 (IQR, 280–1114; minimum, 118; maximum, 1825) days.

### 3.2. Post-Operative Complications

We analyzed post-postoperative complications which occurred during the follow-up period (Table S1). In the entire cohort, vascular complication (7.7% in the antiplatelet group vs. 8.8% in the non-antiplatelet group, respectively, *p* = 0.895), bleeding (5.3% vs. 8.8%, *p* = 0.311), and bile duct complication (37.0% vs. 32.4%, *p* = 0.496) were not different between two groups. Those complications were similar between the two matched cohort, either (7.6% vs. 6.3%, *p* = 0.999 for vascular complication; 8.9% vs. 8.9%, *p* = 0.999 for bleeding; 39.2% vs. 32.9%, *p* = 0.523 for bile duct complication)

### 3.3. Kaplan–Meier Analyses in the Matched Cohort

Of 158 matched patients, 27 (17.1%) experienced recurrence of HCC, and 32 (20.3%) died during a mean follow-up of 43.9 ± 19.6 months. Among the patients who experienced HCC recurrence, the median time from LT to HCC recurrence was 12.0 months (IQR, 9.1–18.9; Figure S2). Of the 27 recurrences, 20 were extrahepatic (74.1% of recurrence, differences between matched groups are shown in Table S2). Among the patients who died, 21 deaths (13.3%) occurred after HCC recurrence (HCC-specific death) and 11 deaths (7.0%) occurred without HCC recurrence (non-HCC death).

**Figure 2:**
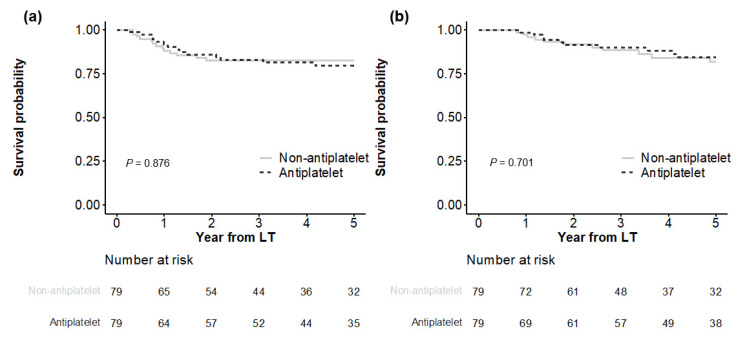
Comparison of outcomes between matched groups. HCC recurrence is shown in (**a**), and HCC-specific death is shown in (**b**).

In Kaplan–Meier analyses (Figure 2), both matched groups showed similar HCC recurrence (*p* = 0.876), with cumulative incidences of 11.9%, 17.5%, and 18.5% at 1, 3, and 5 years in the antiplatelet group versus 8.2%, 14.0%, and 20.4% at 1, 3, and 5 years in the non-antiplatelet group, respectively. HCC-specific deaths were also not significantly different (*p* = 0.701), with cumulative incidences of 2.7%, 11.6%, and 18.3% at 1, 3, and 5 years in the antiplatelet group versus 1.4%, 10.1%, and 15.4% at 1, 3, and 5 years in the non-antiplatelet group, respectively. 

All-cause death did not differ significantly between the matched groups (*p* = 0.867), with cumulative incidences of 5.2%, 15.3%, and 23.5% at 1, 3, and 5 years, respectively, in the antiplatelet group versus 6.4%, 17.4%, and 24.3% at 1, 3, and 5 years, respectively, in the non-antiplatelet group (Figure S3). Non-HCC death was not significantly different between the two groups (*p* = 0.413), with cumulative incidences of 2.6%, 4.2%, and 6.4% at 1, 3, and 5 years, respectively, in the antiplatelet group versus 5.1%, 8.2%, and 10.5% at 1, 3, and 5 years, respectively, in the non-antiplatelet group.

### 3.4. Multivariate Cox Analyses in the Entire Cohort

In the entire cohort, 71 of 468 (15.2%) patients experienced HCC recurrence and 66 patients (14.1%) died during the mean follow-up period of 43.1 ± 20.9 months. Among patients whose HCC recurred, the median time from LT to recurrence was 13.1 (IQR 9.2–23.1) months. Of the 71 recurrences, 52 were extrahepatic (73.2% of recurrence, differences between entire groups are shown in Table S2). Among the patients who died, 45 deaths (9.6%) occurred after HCC recurrence (HCC-specific death) and 21 deaths (4.5%) occurred without HCC recurrence (non-HCC death).

As shown in Table 2, multivariate Cox analyses revealed that antiplatelet use was not associated with HCC recurrence when treating non-HCC death as a competing risk (hazard ratio [HR], 1.37; 95% confidence interval [CI], 0.76–2.48; *p* = 0.300). Other factors associated with HCC recurrence were age (HR. 0.94; *p* = 0.008), diabetes (HR, 0.57; *p* = 0.047), pre-transplant MELD (HR, 0.93; *p* = 0.027), AFP by log scale (HR, 1.18; *p* = 0.022), bridging therapy (HR, 0.10; *p* < 0.001 for none and HR, 2.14; *p* = 0.028 for systemic versus locoregional), viable tumor number (HR, 1.06; *p* = 0.031), maximum tumor size (HR, 1.18; *p* = 0.003), microvascular invasion (HR, 2.45; *p* = 0.001), and platelet-lymphocyte ratio (HR, 1.00; *p* = 0.013).

Antiplatelet therapy was not a significant protective factor for HCC-specific death in the competing risk regression model (HR, 1.54; 95% CI, 0.67–3.56; *p* = 0.310). Other factors associated with HCC-specific death were donor age (HR, 1.03; *p* = 0.031), AFP by log scale (HR, 1.10; *p* = 0.003), bridging therapy (HR, 0.13; *p* = 0.002 for none and HR, 3.39; *p* = 0.006 for systemic versus locoregional), viable tumor number (HR, 1.08; *p* < 0.001), satellite nodule (HR, 4.96; *p* < 0.001), and immunosuppressants (HR, 0.29; *p* = 0.004 for tacrolimus/mTOR inhibitor versus tacrolimus). In the analyses of secondary outcomes, antiplatelet use was not associated with all-cause death (HR, 1.54; 95% CI, 0.67–3.56; *p* = 0.310; Table S3) but also with non-HCC death (HR, 0.91; 95% CI, 0.19–4.30; *p* = 0.900; Table S4). 

## 4. Discussion

This observational study evaluated the association between antiplatelet treatment and outcomes in HCC patients who underwent LT. To assure the independent effect of antiplatelet therapy, we explored a matched cohort and analyzed the entire cohort by adjusting covariates with all available variables including explant pathology. Furthermore, to control the effect of death resulting from causes other than HCC in LT patients, competing risk regression was applied for analyses of HCC-related outcomes, such as HCC recurrence and HCC-specific death. Using these reliable approaches, we found that antiplatelet treatment did not affect HCC recurrence or HCC-related mortality in the LT recipients. Inhibition of cyclooxygenase (COX)-2, nuclear factor-kB signaling, and inhibition of protein kinase 3 have been suggested as the mechanisms of the tumor suppression effect of aspirin [17,18,19]. In addition, downregulation of the P2Y12 adenosine diphosphate receptor resulting in the restriction of platelet activity has been suggested for clopidogrel action on tumors [20]. The antitumor effects of antiplatelet agents have been broadly investigated in colorectal cancer with positive results, especially in subgroups with the expression of COX-2 and mutated PIK3CA genes [21]. In addition to these effects, patients with HCC usually accompanied by underlying liver disease could benefit from the additional anti-inflammatory effects of antiplatelet agents via various mechanisms, such as preventing the accumulation of CD8+ T-lymphocytes [7] and platelet-derived growth factor-β [22], resulting in reduced fibrosis and HCC.

However, the suppressive effect of antiplatelet agents on HCC recurrence was not observed in our LT-only population. We hypothesized this discrepancy with previous studies with non-LT populations. First, many more confounding factors affect HCC outcomes in LT patients than in the non-LT population. Aside from pre-transplant treatment, underlying disease, tumor markers, explant pathology, and post-transplant management, such as immunosuppression, might vary across patients and could affect HCC outcomes more than the effect of antiplatelet [23]. Another reason could be that LT treats HCC as well as the underlying liver disease that causes cirrhosis. After explanting the native liver and implanting the graft, antiplatelet therapy slightly affected inflammation in the liver, resulting in the progression of cirrhosis. For example, hepatitis B patients, accounting for more than 70% of our study population, usually receive antihepatitis B immunoglobulin with or without oral nuclos(t)ide in Korea, resulting in an extremely low rate of disease recurrence after LT [24]. 

The duration of antiplatelet exposure could be another important issue in this study compared to previous studies in patients with liver cirrhosis. In a recent Korean population-based study, aspirin use was defined as more than 3 years of aspirin prescription during 7.5 years of mean follow-up [25]. In another Swedish population-based study [9], aspirin use was defined as more than 90 days of prescription, which was the same as in this study; however, the mean follow-up was 7.9 years, which was much longer than that in this study. Furthermore, they reported that the relationship between aspirin and the development of HCC was duration dependent. In our LT population, the median time from LT to HCC recurrence was 12 months, and most recurrences occurred within 3 years (Figure S2). The relatively short duration of antiplatelet use with respect to the timing of HCC recurrence could have affected the negative results of our study. Further studies are needed to determine whether antiplatelet use for a longer duration can affect oncologic outcomes in LT patients.

To our knowledge, only one population-based study has evaluated the effect of aspirin in patients who received specific treatments for HCC [11]. They demonstrated that antiplatelet therapy significantly reduced HCC recurrence and overall mortality after resection from matched analysis, although the mean duration of antiplatelet therapy (1.3 years) and median follow-up (3.9 years) were shorter than those in our study population. In addition to the contribution of cirrhosis in the remnant liver after resection, we hypothesized the discrepancy in our results derived from the absence of critical information in that study, such as pre-operational treatment, tumor markers, or pathologic features, which is an inevitable weakness of population-based studies. In contrast, our study, using detailed institutional data, could provide more reliable results regarding the association between antiplatelet therapy and HCC prognosis after adjusting for important covariates in LT patients. Further studies are needed in patients who undergo specific treatment for HCC, containing critical information related to HCC recurrence.

Among other factors, mTOR inhibitor appeared as a significant protective factor for HCC-specific death, although it did not reduce HCC recurrence itself in the current study. mTOR inhibitor is known for its anticancer effect via decreasing the production of vascular endothelial growth factor and inhibiting vascular endothelial cell response to it [26]. The largest randomized controlled trial for the clinical effect of mTOR inhibitor in LT recipients for HCC recently showed mTOR inhibitor used for more than 3 months significantly reduced overall survival [27], although it failed to show a preventive effect for HCC recurrence in its initial analyses [28]. Our results were in line with those studies, indicating that mTOR inhibitors could not prevent HCC recurrence themselves but attenuated the aggressiveness of recurred tumors. This needs further study, which is specifically designed.

The relatively small sample size, single-center, and retrospective design were the limitations of our study. However, the recruitment of numerous variables associated with LT and HCC prognosis was a strength of this study, which was not performed in a large number of population-based studies. The heterogeneous indication for antiplatelet use in our study population was another limitation to hinder an intention-to-treat approach. Lastly, the relatively short duration of antiplatelet use before primary endpoints in our LT subjects might have made our results differ from those of prior studies with non-LT subjects whose antiplatelet use had a longer duration. 

## 5. Conclusions

This study was the first to investigate the effect of antiplatelet therapy on the prognosis of HCC patients after LT. Unlike usual settings with cirrhosis or hepatitis, we found that antiplatelet therapy did not affect HCC recurrence or HCC-specific mortality when used after LT. Further studies with larger sample size and intentional purposes should be implemented to clarify the relationship between antiplatelet therapy and HCC in LT patients.

## Figures and Tables

**Table 1:** Baseline characteristics.

	Before Matching			After Matching		
Variables	Antiplatelet (*n* = 377)	Non-Antiplatelet (*n* = 91)	*p*	Antiplatelet (*n* = 79)	Non-Antiplatelet (*n* = 79)	SMD
Age, year	55 (51–61)	55.0 (51–59)	0.977	57 (52–62)	55 (51–59)	0.026
Sex, female	67 (17.8)	20 (22.0)	0.438	15 (19.0)	17 (21.5)	0.061
BMI, kg/m^2^	24.2 (22.5–26.2)	23.8 (21.9–26.0)	0.298	23.8 (22.1–26.6)	24.1 (22.1–26.0)	0.043
Year of LT			0.073			0.156
2005–2014	187 (49.6)	35 (38.5)		27 (34.2)	33 (41.8)	
2015–2021	190 (50.4)	56 (61.5)		52 (65.8)	46 (58.2)	
Underlying liver disease			0.059			0.081
HBV	306 (81.2)	62 (68.1)		59 (74.7)	56 (70.9)	
HCV	23 (6.1)	9 (9.9)		11 (13.9)	6 (7.6)	
Alcohol	28 (7.4)	12 (13.2)		4 (5.1)	11 (13.9)	
Others	20 (5.3)	8 (8.8)		5 (6.3)	6 (7.6)	
ABO incompatibility	54 (14.3)	19 (20.9)	0.166	19 (24.1)	16 (20.3)	0.093
Hypertension	90 (23.9)	23 (25.3)	0.885	13 (16.5)	20 (25.3)	0.104
Diabetes	105 (27.9)	31 (34.1)	0.297	20 (25.3)	26 (32.9)	0.160
Cardiovascular disease	28 (7.4)	5 (5.5)	0.676	2 (2.5)	5 (6.3)	0.167
Pre-transplant MELD	10 (8–14)	12 (8–16)	0.023	13 (9–17)	12 (8–16)	0.073
Donor type			0.917			0.064
Living	298 (79.0)	73 (80.2)		61 (77.2)	63 (79.7)	
Deceased	79 (21.0)	18 (19.8)		18 (22.8)	16 (20.3)	
Donor age, year	34 (26–45)	31 (24–43)	0.333	31 (25–41)	31 (24–43)	0.022
Donor sex, female	149 (39.5)	27 (29.7)	0.105	20 (25.3)	24 (30.4)	0.111
Graft steatosis >10%	28 (7.4)	2 (2.2)	0.112	4 (5.1)	2 (2.5)	0.173
AFP, ng/mL	7.3 (3.5–25.0)	7.5 (3.4–29.2)	0.973	8.2 (4.1–29.7)	7.3 (3.4–25.4)	0.028
PIVKA II, mAU/mL	35 (20–97)	37 (21–111)	0.598	38 (21–97)	40 (21–114)	0.081
Salvage LT	52 (13.8)	11 (12.1)	0.797	9 (11.4)	11 (13.9)	0.078
Bridging therapy			0.618			0.058
Locoregional	231 (61.3)	56 (61.5)		51 (64.6)	51 (64.6)	
None	108 (28.6)	23 (25.3)		21 (26.6)	19 (24.1)	
Systemic	38 (10.1)	12 (13.2)		7 (8.9)	9 (11.4)	
Explant pathology						
Total necrosis	56 (14.9)	15 (16.5)	0.821	13 (16.5)	13 (16.5)	<0.001
Viable tumor number	1.0 (1.0–3.0)	1.0 (1.0–3.0)	0.419	1.0 (1.0–3.0)	1.0 (1.0–2.5)	0.157
Maximum tumor size	1.8 (1.1–2.8)	1.8 (1.0–2.8)	0.812	1.6 (1.2–2.5)	1.7 (1.0–2.8)	0.094
Sum of tumor size	2.5 (1.1–4.7)	2.3 (1.0–5.5)	0.635	2.4 (1.2–4.6)	2.3 (1.0–5.5)	0.016
Microvascular invasion	87 (23.1)	20 (22.0)	0.932	19 (24.1)	20 (25.3)	0.031
Poor differentiation	107 (28.4)	30 (33.0)	0.463	31 (39.2)	25 (31.6)	0.162
Satellite nodule	37 (9.8)	7 (7.7)	0.673	4 (5.1)	6 (7.6)	0.095
Hospital stay, days	22 (18–29)	26 (21–41)	0.001	23 (18–34)	26 (20–36)	0.156
Immunosuppressants ^a^			0.576			0.159
Tacrolimus	128 (34.0)	26 (28.6)		14 (17.7)	22 (27.8)	
Tacrolimus +Mycophenolate	129 (34.2)	32 (35.2)		35 (44.3)	29 (36.7)	
Tacrolimus +mTOR inhibitor	120 (31.8)	33 (36.3)		30 (38.0)	28 (35.4)	
Platelet count, k/μL	77 (56–108)	71 (51–94)	0.078	68 (52–93)	71 (51–96)	0.161
Platelet-lymphocyte ratio	2.9 (1.8–4.6)	2.8 (2.0–5.4)	0.923	3.0 (2.0–4.4)	2.8 (2.0–5.7)	0.152
Neutrophil-lymphocyte ratio	100.0 (70.5–140.7)	95.4 (76.7–137.7)	0.820	90.2 (60.6–122.4)	95.4 (76.7–134.3)	0.134
Laboratory results for liver function at 1 month						
AST, U/L	25 (17–40)	24 (15–40)	0.572	26 (19–43)	23 (15–37)	0.046
ALT, U/L	27 (15–57)	25 (17–63)	0.985	27 (15–54)	25 (17–60)	0.021
Total bilirubin, mg/dL	0.8 (0.6–1.0)	0.8 (0.6–1.2)	0.542	0.8 (0.6–1.1)	0.8 (0.6–1.2)	0.051
INR	1.0 (0.9–1.0)	1.0 (0.9–1.1)	0.003	1.0 (0.9–1.0)	1.0 (0.9–1.1)	0.033

^a^ Immunosuppressants were categorized according to regimens used at 3 months after LT. BMI, body mass index; HBV, hepatitis B virus; HCV, hepatitis C virus; LT, liver transplantation; MELD, modified end-stage liver disease; SMD, standardized mean difference.

**Table 2:** Multivariable analyses for HCC recurrence and HCC-specific death.

	HCC Recurrence ^a^			HCC-Specific Death ^a^		
Variables	HR	95% CI	*p*	HR	95% CI	*p*
Antiplatelet group						
Non-antiplatelet	1.00			1.00		
Antiplatelet	0.73	0.40–1.32	0.300	0.65	0.28–1.49	0.310
Age	0.94	0.90–0.98	0.008			
Diabetes	0.57	0.33–0.99	0.047			
Cardiovascular disease				1.69	0.45–6.29	0.430
Donor age, year	1.01	0.99–1.03	0.160	1.03	1.00–1.05	0.031
Log AFP	1.18	1.0–1.37	0.022	1.33	1.10–1.59	0.003
Bridging therapy						
Locoregional	1.00			1.00		
None	0.10	0.03–0.35	<0.001	0.13	0.04–0.48	0.002
Systemic	2.14	1.09–4.22	0.028	3.39	1.41–8.13	0.006
Viable tumor number	1.06	1.01–1.11	0.031	1.08	1.05–1.12	<0.001
Maximum tumor size	1.18	1.06–1.32	0.003			
Microvascular invasion	2.45	1.42–4.23	0.001			
Poor differentiation				1.96	0.93–4.11	0.075
Satellite nodule	1.69	0.94–3.03	0.080	4.96	2.29–10.7	<0.001
Hospital stay	1.02	1.00–1.04	0.100	1.03	0.99–1.05	0.058
Immunosuppressants						
Tacrolimus				1.00		
Tacrolimus +Mycophenolate				0.49	0.23–1.04	0.063
Tacrolimus +mTOR inhibitor				0.29	0.12–0.67	0.004
Platelet-lymphocyte ratio	1.00	1.00–1.01	0.013			
AST at 1 month, U/L	1.00	1.00–1.01	0.280	1.00	1.00–1.01	0.093

^a^ Multivariable analyses were performed treating non-HCC death as competing risk. HCC, hepatocellular carcinoma; LT, liver transplantation; MELD, modified end-stage liver disease.

## Data Availability

The data presented in this study are available in this article and supplementary material.

## Supplementary Materials

The following supporting information can be downloaded at: https://www.mdpi.com/article/10.3390/cancers14215329/s1, Figure S1: Serial tacrolimus trough level during study period; Table S1: Comparison of postoperative complications in the entire and in the matched cohorts; Figure S2: Distribution of months from LT to HCC recurrence in matched cohort; Table S2: Patterns of HCC recurrence in the entire and in the matched cohorts; Figure S3: Comparison of all-cause death and non-HCC death between matched groups; Table S3: Multivariable Cox regression analyses for all-cause death; Table S4: Multivariable Cox regression analyses for non-HCC death.
