# Supplementary material for: Antiplatelet Drugs on the Recurrence of Hepatocellular Carcinoma after Liver Transplantation
Source: Cancers (Basel). 2022 Oct 29;14(21):5329. doi: 10.3390/cancers14215329 (PMC9654602; doi:10.3390/cancers14215329)
Supplement: Supplementary file 1 [file cancers-14-05329-s001.zip › cancers-1965191-supplementary.pdf]

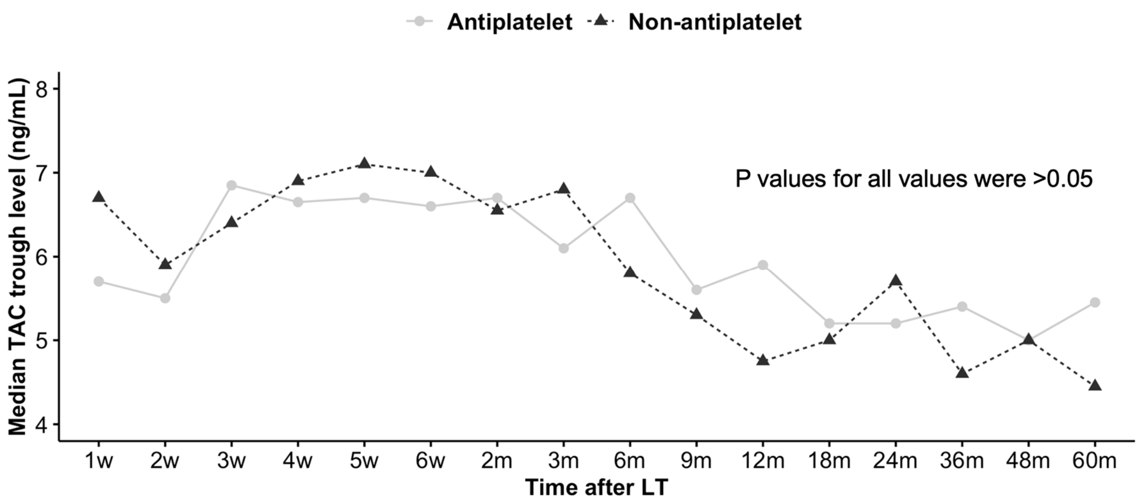

Figure S1. Serial tacrolimus trough level during study period.

Table S1. Comparison of postoperative complications in the entire and in the matched cohorts.

	**Antiplatelet, entire (n=91)**	**Non-antiplatelet, entire (n=377)**	***P***
**Vascular complication**	29 (7.7%)	8 (8.8%)	0.895
**Bleeding**	20 (5.3%)	8 (8.8%)	0.311
**Bile duct complication**	30 (37.0%)	122 (32.4%)	0.496
	**Antiplatelet, matched (n=79)**	**Non-antiplatelet, matched (n=79)**	***P***
**Vascular complication**	6 (7.6%)	5 (6.3%)	0.999
**Bleeding**	7 (8.9%)	7 (8.9%)	0.999
**Bile duct complication**	31 (39.2%)	23 (32.9%)	0.523

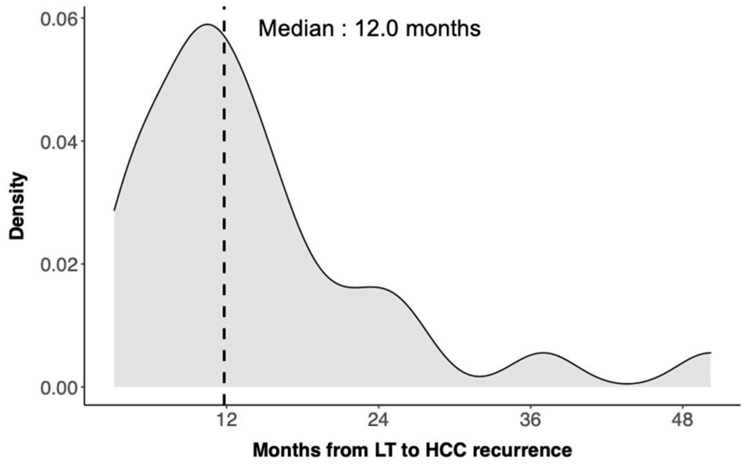

Figure S2. Distribution of months from LT to HCC recurrence in matched cohort

Table S2. Patterns of HCC recurrence in the entire and in the matched cohorts.

	**Antiplatelet, entire (n=16)**	**Non-antiplatelet, entire (n=55)**
**Intrahepatic recurrence**	5 (31.2%)	14 (25.5%)
**Extrahepatic recurrence**	11 (68.8%)	41 (74.5%)
	**Antiplatelet, matched (n=13)**	**Non-antiplatelet, matched (n=14)**
**Intrahepatic recurrence**	3 (23.1%)	4 (28.6%)
**Extrahepatic recurrence**	10 (76.9%)	10 (71.4%)

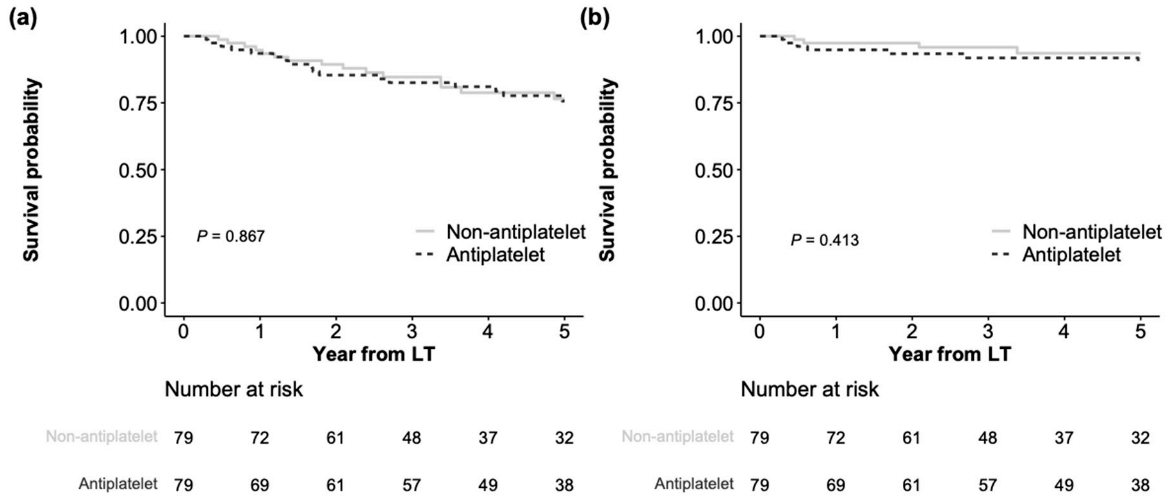

Figure S3. Comparison of all-cause death and non-HCC death between matched groups.

(a) all-cause death, (b) non-HCC death

Table S3. Multivariable Cox regression analyses for all-cause death.

Characteristic	HR[1]	95% CI[1]	p-value
Antiplatelet_groupAntiplatelet	0.75	0.41, 1.38	0.360
Age	1.04	0.99, 1.10	0.120
SexFemale	0.54	0.25, 1.14	0.110
HypertensionYes	0.58	0.31, 1.10	0.095
MELD	0.94	0.90, 0.99	0.010
Donor_typeDeceased	1.78	0.90, 3.52	0.098
Donor_age	1.03	1.01, 1.05	0.013
Graft_steatosis>10%	0.40	0.11, 1.46	0.170
log_ AFP	1.20	1.03,1.41	0.024
Bridging_therapyNone	0.51	0.23, 1.12	0.094
Bridging_therapySystemic	3.51	1.92, 6.41	<0.001
Viable_tumor_number	1.08	1.05, 1.11	<0.001
Satellite_noduleYes	3.70	1.86, 7.35	<0.001
Hospital_stay	1.04	1.02, 1.05	<0.001
ImmunosuppressantsTAC+MMF	0.98	0.58, 1.66	0.940
ImmunosuppressantsTAC+mTORi	0.41	0.20, 0.86	0.019
AST_m1	1.01	1.00, 1.01	<0.001

1 HR = Hazard Ratio, CI = Confidence Interval

Table S4. Multivariable Cox regression analyses for non-HCC death.

HR was calculated by Fine and Gray method treating HCC-death as competing risk.

Characteristic	HR[1]	95% CI[1]	p-value
Antiplatelet_ groupAntiplatelet	1.10	0.23, 5.23	0.900
Age	1.17	1.09, 1.26	<0.001
Year_of_LT2015-2021	0.24	0.07, 0.82	0.023
ABO_ jincompablityYes	6.99	0.90, 54.3	0.063
Diabetes_ mellitusYes	2.53	0.87, 7.38	0.089
Donor_ age	1.05	1.00, 1.11	0.055
Bridging_therapyNone	2.56	0.89, 7.37	0.081
Bridging_therapySystemic	9.61	2.37, 39.0	0.002
Sum_of_tumor_size	0.80	0.66, 0.97	0.023
Microvascular_invasionYes	0.08	0.00, 3.18	0.180
Hospital_stay	1.05	1.02, 1.09	0.002
ImmunosuppressantsTAC+MMF	4.36	1.34, 14.2	0.015
ImmunosuppressantsTAC+mTORi	0.87	0.08, 9.20	0.910
Neutrophil_lymphocyte_ratio	0.75	0.65, 0.87	<0.001
AST_m1	1.01	1.00, 1.02	<0.001
Total_biliubin_m1	1.04	1.02, 1.07	0.002

1 HR = Hazard Ratio, CI = Confidence Interval
